# Supplementary material for: HIVprotI: an integrated web based platform for prediction and design of HIV proteins inhibitors
Source: J Cheminform. 2018 Mar 9;10:12. doi: 10.1186/s13321-018-0266-y (PMC5845081; doi:10.1186/s13321-018-0266-y)
Supplement: Supplementary file 2 — Additional file 2. Source code of HIVProtI web server. [file 13321_2018_266_MOESM2_ESM.zip › HIVprotI_Source-code/hivprotI/weka-3-6-6/doc/weka/core/neighboursearch/package-tree.html]

weka.core.neighboursearch Class Hierarchy


---


|  |  |  |
| --- | --- | --- |
| **Overview** \| **Package** \| Class \| **Tree** \| **Deprecated** \| **Index** \| **Help** \| **Weka's home** | |  |
| **PREV**   **NEXT** | **FRAMES**    **NO FRAMES**     **All Classes** |  |


---


## Hierarchy For Package weka.core.neighboursearch

**Package Hierarchies:**: All Packages

---

## Class Hierarchy

- java.lang.Object
  - weka.core.neighboursearch.**CoverTree.CoverTreeNode** (implements weka.core.RevisionHandler, java.io.Serializable)- weka.core.neighboursearch.**NearestNeighbourSearch** (implements weka.core.AdditionalMeasureProducer, weka.core.OptionHandler, weka.core.RevisionHandler, java.io.Serializable)
      - weka.core.neighboursearch.**BallTree** (implements weka.core.TechnicalInformationHandler)- weka.core.neighboursearch.**CoverTree** (implements weka.core.TechnicalInformationHandler)- weka.core.neighboursearch.**KDTree** (implements weka.core.TechnicalInformationHandler)- weka.core.neighboursearch.**LinearNNSearch**
      - weka.core.neighboursearch.**PerformanceStats** (implements weka.core.AdditionalMeasureProducer, weka.core.RevisionHandler, java.io.Serializable)
        - weka.core.neighboursearch.**TreePerformanceStats**

---


|  |  |  |
| --- | --- | --- |
| **Overview** \| **Package** \| Class \| **Tree** \| **Deprecated** \| **Index** \| **Help** \| **Weka's home** | |  |
| **PREV**   **NEXT** | **FRAMES**    **NO FRAMES**     **All Classes** |  |


---
